# Identification of Methylation-Regulated Differentially Expressed Genes and Related Pathways in Hepatocellular Carcinoma: A Study Based on TCGA Database and Bioinformatics Analysis

**DOI:** 10.3389/fonc.2021.636093

**Published:** 2021-06-03

**Authors:** Yu Liang, Bin Ma, Peng Jiang, Hong-Mei Yang

**Affiliations:** ^1^ Department of Colorectal Surgery, Cancer Hospital of China Medical University, Liaoning Cancer Hospital & Institute, Shenyang, China; ^2^ Department of Internal Oncology, Cancer Hospital of China Medical University, Liaoning Cancer Hospital & Institute, Shenyang, China

**Keywords:** hepatocellular carcinoma, DNA methylation, overall survival, bioinformatic analysis, methylation-regulated differentially expressed genes

## Abstract

**Background:**

In recent years, DNA methylation modification has been shown to be a critical mechanism in the field of epigenetics.

**Methods:**

Hepatocellular carcinoma (HCC) data were obtained from The Cancer Genome Atlas project, including RNA expression profiles, Illumina Human Methylation 450K BeadChip data, clinical information, and pathological features. Then, differentially expressed genes (DEGs) and differentially methylated genes were identified using R software. Methylation-regulated DEGs (MeDEGs) were further analyzed using Spearman’s correlation analysis. Gene ontology (GO) and Kyoto Encyclopedia of Genes and Genomes (KEGG) pathway analyses were performed using the DAVID database and ClueGO in Cytoscape software. Kaplan–Meier survival analysis explored the relationship between methylation, expression of MeDEGs, and survival time. Gene set enrichment analysis (GSEA) was conducted to predict the function of prognosis-related MeDEGs.

**Results:**

A total of nine up-regulated and 72 down-regulated MeDEGs were identified. GO and KEGG pathway analyses results indicated that multiple cancer-related terms were enriched. Kaplan–Meier survival analysis showed that the methylation status of four MeDEGs (CTF1, FZD8, PDK4, and ZNF334) was negatively associated with overall survival. Moreover, the methylation status of CDF1 and PDK4 was identified as an independent prognostic factor. According to GSEA, hypermethylation of prognosis-related MeDEGs was enriched in pathways that included “Spliceosome”, “Cell cycle”, “RNA degradation”, “RNA polymerase”, “DNA replication”, “Mismatch repair”, “Base excision repair”, “Nucleotide excision repair”, “Homologous recombination”, “Protein export”, and “Pyrimidine metabolism”.

**Conclusions:**

Aberrant DNA methylation plays a critical role in malignant progression of HCC. Prognosis-related MeDEGs identified in this research may be potential biomarkers and targets in diagnosis and treatment.

## Introduction

Hepatocellular carcinoma (HCC) is the most common histopathological type of liver cancer, which ranked seventh in incidence and third in mortality among tumors worldwide in 2018 (1). Epidemiological studies have confirmed that HCC occurrence is associated with chronic hepatitis B/C virus infection, liver cirrhosis, environmental toxins, non-alcoholic fatty liver disease, metabolic disease, and lifestyle factors (2, 3). Although surgery combined with chemotherapy, radiotherapy, and immunotherapy can improve patient prognosis, the five-year survival rate in advanced-stage patients is still < 15% (4). Hence, a study into crucial biomarkers and molecular therapeutic pathways is of great significance for improving HCC patient prognosis.

DNA methylation modification has been considered to be a critical gene regulation mechanism in epigenetics and has been verified to be a reversible process. In the genome of normal cells, promoter cytosine-phosphate-guanine (CpG)-islands are typically hypomethylated. However, tumor cell hypermethylation of the CpG-island in the tumor suppressor promoter region is associated with malignant formation and progression. For instance, zinc finger protein 382 (ZNF382) is a potent tumor-suppressor and is down-regulated in hepatitis B-related HCC due to promoter methylation (5). However, research into DNA methylation of individual genes and pathways remains insufficient. Screening methylation-regulated differentially expressed genes (MeDEGs) with high-throughput data is of profound significance for clarifying the role of methylation and identifying future research directions.

In recent years, diverse gene-sequencing platforms have been utilized in basic and clinical HCC research. In addition, these techniques provide evidence for accurate tumor therapy. For instance, Illumina Human Methylation 450K BeadChip has been employed to detect genome‐wide aberrant DNA methylation profiles between HCC cell line Huh7 and normal cell line L02. As a result, 62,702 (61.3%) CpG-island sites were hypermethylated and 39,552 (38.7%) CpG-island sites were hypomethylated (6). Zhang et al. have indicated that distinct DNA methylation differences emerge in the host immune system at an early stage based on the Illumina Human Methylation 450K BeadChip data, which may serve as noninvasive diagnostic HCC markers (7). The Illumina Methylation 450K BeadChip has been shown to play a critical role in the field of tumor epigenetics, but there is still a lack of conjoint correlation analysis of methylation, gene expression, and patient prognosis in large cohorts.

The present study applied bioinformatics analysis to identify MeDEGs based on *in silico* and clinical data from The Cancer Genome Atlas (TCGA, http://cancergenome.nih.gov) project (8). Then, MeDEG enrichment analysis was performed using an online database. Methylation of four genes was associated with prognosis in HCC patients. Gene set enrichment analysis (GSEA) was also performed.

## Materials and Methods

### Data Collection and MeDEG Identification

TCGA database included the expression profiles of 374 HCC and 50 normal tissues (level 3) derived using RNA-seq and methylation data from 380 HCC and 50 normal tissues analyzed with the Illumina Human Methylation 450K BeadChip platform up to March 2020. Genomic Data Commons Data Transfer Tool 1.3.0 (8) was used to download the above profiles and clinical information data for further analysis. This research conformed to the guidelines published by TCGA on December 2015 (https://cancergenome.nih.gov/publications/publicationguidelines) and approval from an ethics committee was not required.

An RNA matrix that included 50 normal hepatic tissues and corresponding HCC tissues was constructed using PERL software. Methylation data matrix including 50 paired HCC and normal tissue samples was constructed using the same method. Differentially expressed genes (DEGs) and differentially methylated genes (DMGs) were identified using the “edgeR” and “limma” packages in R software with a threshold log2 fold change (FC) > 1.0 and P < 0.01. After a total of 42 normal and 374 HCC tissues were analyzed using RNA-seq and Illumina Human Methylation 450K BeadChip platform, expression and methylation data were merged together for Spearman’s correlation analysis. The hypermethylated down-regulated and hypomethylated up-regulated genes that satisfied the cut-off criteria, including correlation coefficient < 0.2 and P < 0.01, were identified as MeDEGs. Furthermore, a heat map of the top 100 differentially expressed and methylated genes in 50 paired tissues were mapped using the “heatmap” package in R software.

### MeDEG Enrichment Analyses

To further clarify the function of MeDEGs in HCC carcinogenesis and progression, gene ontology (GO) (9) and Kyoto Encyclopedia of Genes and Genomes (KEGG) pathways (10) analyses were performed using the DAVID database (https://david.ncifcrf.gov/) and ClueGO (11, 12) in Cytoscape 3.7.1. The enrichment results of GO and KEGG analyses were visualized as a bubble chart and network diagram, respectively. Differences with P < 0.05 were regarded as statistically significant.

### Association Analysis of MeDEGs and Patient Prognosis

A total of 353 enrolled HCC patients were followed up for 80 months and had complete clinical data for the survival analysis. The 353 HCC patients were sorted into two groups according to the MeDEG median methylation value. In addition, a hypermethylation and low-expression MeDEG (Hyper-LG) group and a hypomethylation and high-expression MeDEG (Hypo-HG) group were established according to the median value of MeDEG methylation and expression. Kaplan–Meier method and log-rank test were used to compare the overall survival between the two groups using the “survival” package in R software. Differences with P < 0.05 were regarded as statistically significant.

### GSEA of Prognosis-Related MeDEGs

GSEA of prognosis-related MeDEGs was conducted using GSEA 3.0 software with gene set c2 (cp.kegg.v.6.2.symbols.gmt). RNA expression profiles for 374 HCC tissues were selected as the dataset. The sample was marked as either “Hypermethylation” or “Hypomethylation” based on the median methylation value of prognosis-related MeDEGs. The enrichment score > 0.4 and P < 0.05 were regarded as statistically significant.

### Statistical Analysis

All statistical analyses were performed using SPSS V18.0 (SPSS, Inc., Chicago, IL, USA). The association between methylation of prognosis-related MeDEGs and clinicopathological characteristics was analyzed using the chi-squared test. Cox proportional hazards model was applied to evaluate the influence of clinical data and methylation on prognosis. Differences with P < 0.05 were regarded as statistically significant.

## Results

### Identification of MeDEGs in HCC

**Figure 1:**
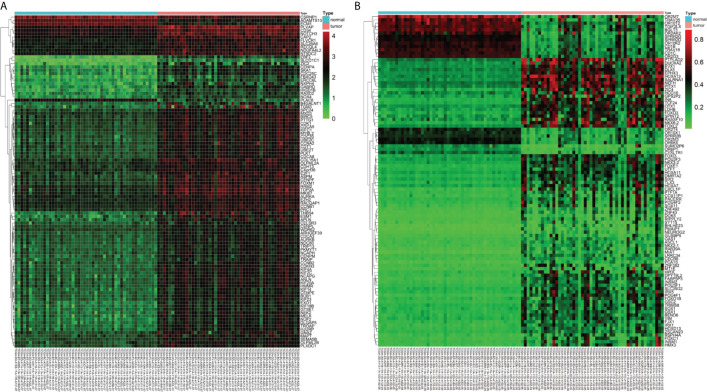
Differentially expressed genes (DEGs) and differentially methylated genes (DMGs) identified from The Cancer Genome Atlas (TCGA) database. **(A)** Heat map of the top 100 DEGs (log_2_ FC > 2, *P* < 0.01). Lower horizontal axis marks sample names, left vertical axis shows clusters of DEGs, and right vertical axis represents gene names. Red represents up-regulated genes and green represents down-regulated genes. **(B)** Heat map of the top 100 DMGs (log_2_ FC > 1, *P* < 0.01). Lower horizontal axis marks sample names, left vertical axis shows clusters of DMGs, and right vertical axis represents gene names. Red represents hypermethylated genes and green represents hypomethylated genes.

**Figure 2:**
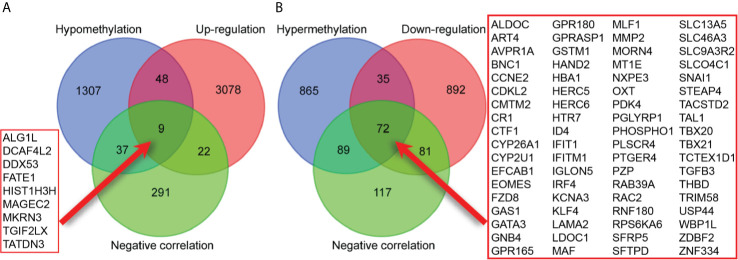
Identification of methylation-regulated differentially expressed genes (MeDEGs). **(A)** A total of nine genes were identified as MeDEGs by intersecting three gene sets (hypomethylation, up-regulation, and negative correlation). **(B)** A total of 72 genes were identified as MeDEGs by intersecting three gene sets (hypermethylation, down-regulation, and negative correlation).

**Figure 3:**
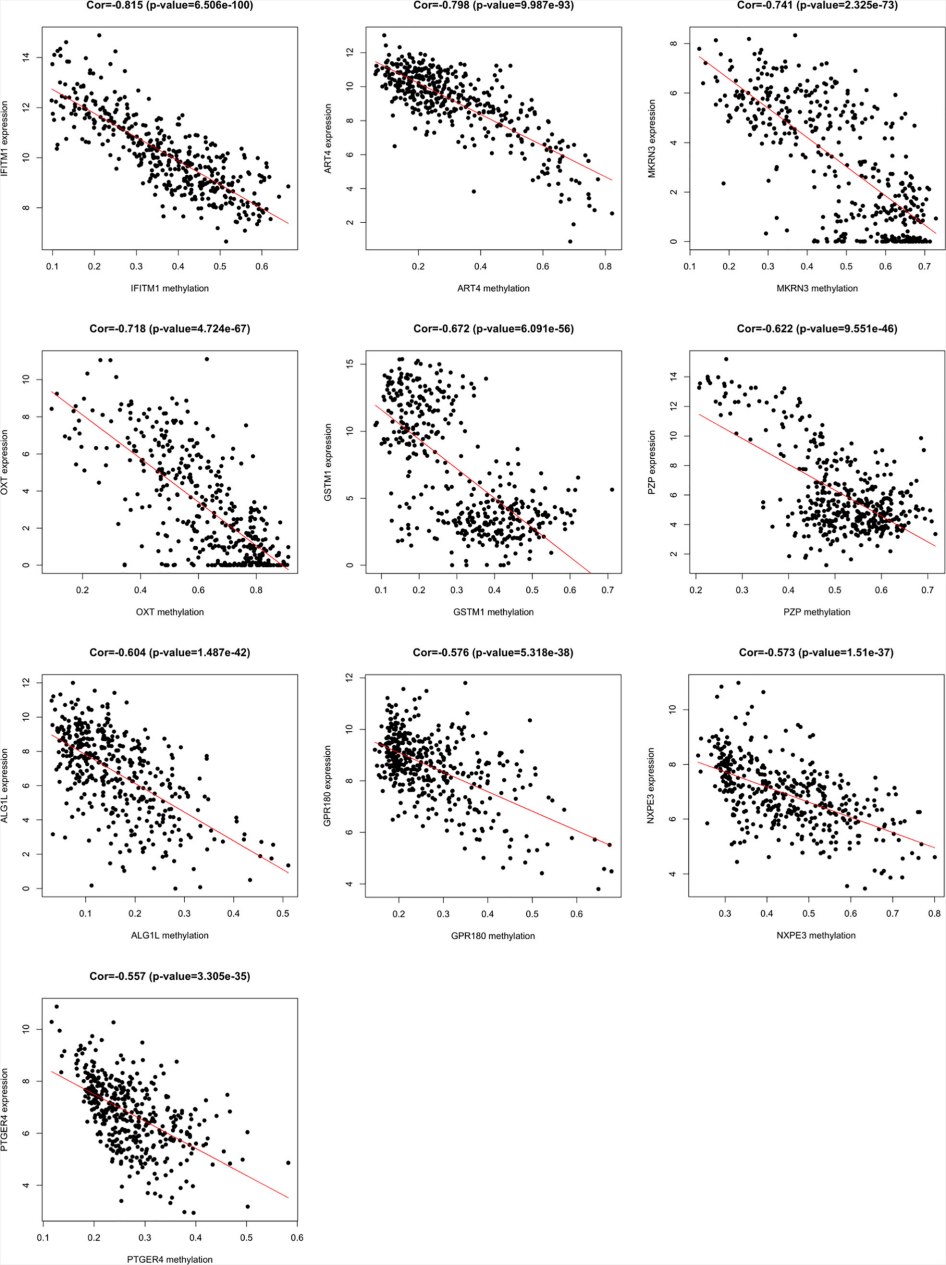
Methylation-regulated differentially expressed genes (MeDEGs) with the top ten correlation coefficients. Spearman’s correlation analysis for methylation (horizontal axis) and expression (vertical axis) of MeDEGs. Spearman’s correlation coefficient and P-values are shown in each plot.

A total of 3157 up-regulated and 1080 down-regulated genes were screened as DEGs from 50 paired HCC and normal tissue samples. The top 100 DEGs with the highest and most significant differences are represented on a heat map in **Figure 1A**. Moreover, 1061 hypermethylated and 1401 hypomethylated DMGs were identified and represented as a heat map of the top 100 DMGs (**Figure 1B**). According to the Spearman’s correlation analysis results, 359 genes had a negative correlation between expression and methylation. Nine up-regulated and 72 down-regulated MeDEGs that satisfied the three conditions were obtained and gene lists were also identified (**Figure 2**). The top ten MeDEGs with the highest Spearman’s correlation coefficient are shown in **Figure 3**.

### Functional Enrichment Analyses of MeDEGs

**Figure 4:**
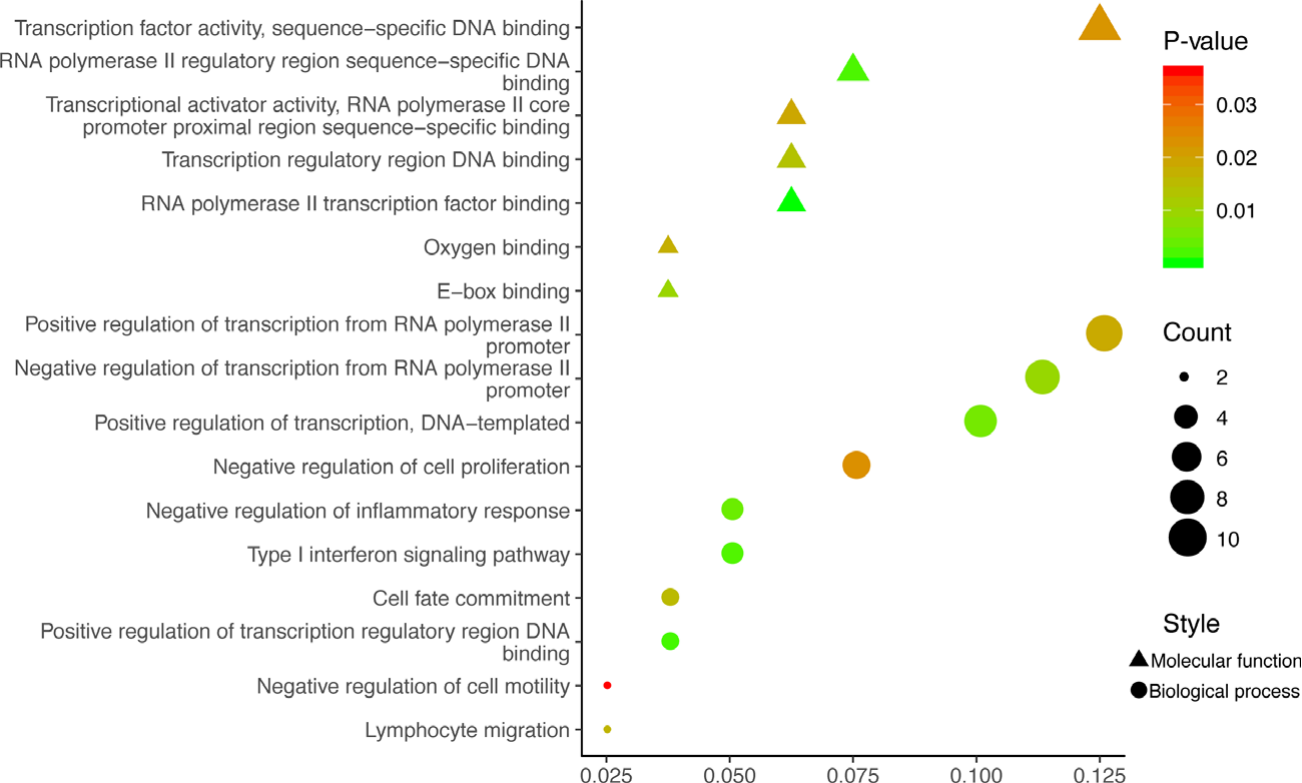
MeDEG gene ontology enrichment analysis. Molecular function and biological process terms for MeDEGs are shown as “triangles” and “circles”, respectively. “Count” represents the number of genes. MeDEGs, methylation-regulated differentially expressed genes.

**Figure 5:**
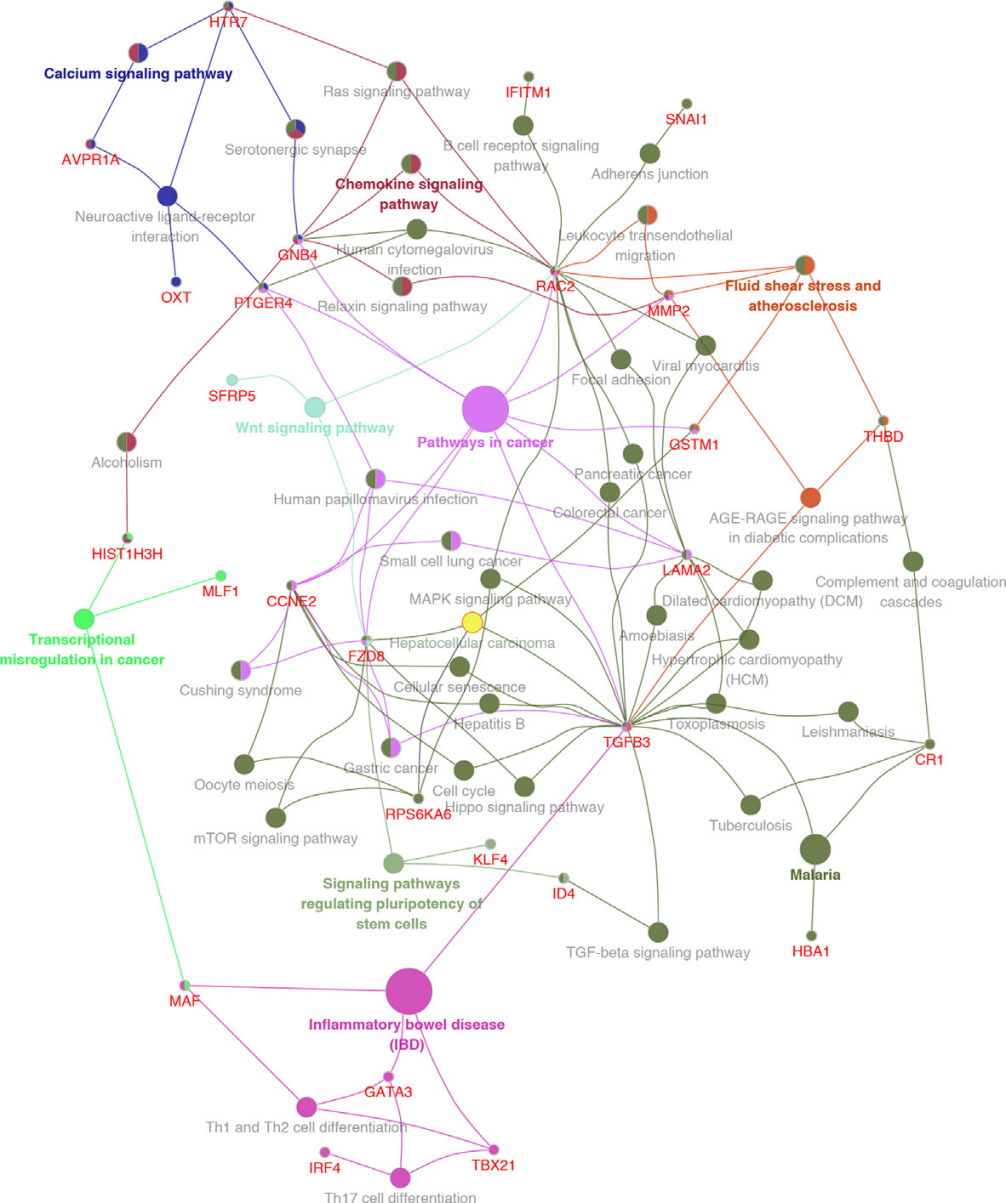
KEGG pathway enrichment network diagram for MeDEGs. MeDEGs and enrichment pathways constitute a regulatory network. The larger the pathway circle, the more genes were enriched.

GO analysis was used to clarify the function of 81 MeDEGs using DAVID 6.8 software (**Figure 4**). The biological process and molecular function terms were mainly associated with transcription regulation. Moreover, negative regulation of cell proliferation and motility was also enriched. In addition, KEGG pathway analysis results indicated that “Pathways in cancer”, “Inflammatory bowel disease”, “Transcriptional misregulation in cancer”, and “Malaria” were significantly involved in MeDEGs. “Hepatocellular carcinoma” was also enriched and KEGG network enrichment diagram was mapped in **Figure 5**.

### Prognosis-Related MeDEGs in HCC

**Figure 6:**
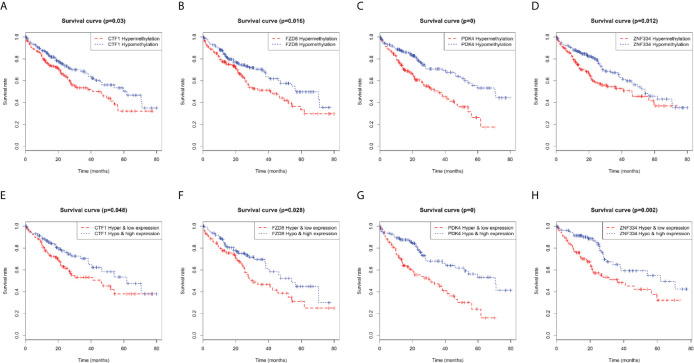
Kaplan-Meier curves for methylation expression of MeDEGs are associated with overall survival. **(A)** CTF1, **(B)** FZD8, **(C)** PDK4 and **(D)** ZNF334 were ranked by the median of methylation and then scored for each patient in accordance with high- or low-level methylation value. **(E)** CTF1, **(F)** FZD8, **(G)** PDK4 and **(H)** ZNF334 were ranked by the median of methylation and expression and then scored for each patient in accordance with high- or low-level methylation value and high or low-level expression value. The horizontal axis represents the overall survival time and the vertical axis represents survival function.

Kaplan–Meier curve analysis revealed a relationship between MeDEG methylation value and overall survival in HCC patients. Hypermethylation of cardiotrophin-1 (CTF1), Frizzled-8 (FZD8), pyruvate dehydrogenase kinase 4 (PDK4), and zinc finger protein 334 (ZNF334) was negatively correlated with the overall survival (**Figures 6A–D**). Then, prognosis of the above four MeDEGs was further compared with the Hyper-LG and Hypo-HG groups. As compared with patients in the Hyper-LG group, Hypo-HG patients had a significant better survival (**Figures 6E–H**).

### Identification of Methylation-Based Biomarkers

A total of 353 patients were divided into “Low” and “High” groups according to the median methylation of CTF1, FZD8, PDK4, and ZNF334. CTF1 methylation status significantly correlated with gender and T stage. In addition, PDK4 hypermethylation was associated with gender, T stage, and pathologic stage (**Table 1**). Univariate and multivariate Cox regression analyses were then conducted to evaluate the prognostic role of the above four genes’ methylation status. The samples were divided into high or low methylation groups according to the median gene methylation status. Advanced T stage, pathologic stage, and high methylation of prognosis-related MeDEGs were associated with poor HCC patient prognosis (**Table 2**). Multivariate analysis results identified T stage and methylation status of CTF1 and PDK4 as independent factors in the overall survival.

**Table 1:** Association between methylation of prognosis-related MeDEGs and clinical features.

Variable	CTF1 methylation		*P*-value	FZD8 methylation		*P-*value	PDK4 methylation		*P-*value	ZNF334 methylation		*P-*value
Age at diagnosis (yr)	Low	High		Low	High		Low	High		Low	High	
<60	89	80	0.366	100	69	0.001**	79	90	0.310	82	87	0.708
≥60	87	97		76	108		97	87		94	90	
Gender												
female	46	65	0.043*	49	62	0.180	42	69	0.003**	55	56	0.971
male	130	112		127	115		134	108		121	121	
T stage												
T1	97	78	0.038*	92	83	0.368	100	75	0.009**	91	84	0.489
T2-4	79	99		84	94		76	102		85	93	
N stage												
N0	173	170	0.202	171	172	0.993	174	169	0.055	170	173	0.741
N1	3	7		5	5		2	8		6	4	
Pathologic stage												
I + II	138	124	0.094	134	128	0.485	140	122	0.031*	133	129	0.564
III + IV	38	53		42	49		36	55		43	48	

*P < 0.05, **P < 0.01.

**Table 2:** Cox regression analyses of association between prognosis-related MeDEGs and clinicopathological characteristics.

Variable	Univariate analysis		Multivariate analysis	
	HR (95%CI)	*P*-value	HR (95%CI)	*P*-value
Age at diagnosis (≥60 *vs* <60)	0.843 (0.582-1.220)	0.365		
Gender (female *vs* male)	0.756 (0.516-1.108)	0.151		
T stage (T2-4 *vs* T1)	2.776 (1.881-3.709)	<0.001**	1.761 (1.283-2.417)	0.003**
N stage (N1 *vs* N0)	1.104 (0.750-1.651)	0.186		
Pathologic stage (III + IV *vs* I + II)	2.122 (1.319-3.471)	<0.001**	1.391 (0.967-2.002)	0.076
CTF1 methylation (high *vs* low)	1.557 (1.055-2.106)	0.022*	1.487 (1.023-2.177)	0.032*
FZD8 methylation (high *vs* low)	1.463 (1.002-2.042)	0.048*	1.245 (0.806-1.838)	0.309
PDK4 methylation (high *vs* low)	1.505 (1.065-2.128)	0.021*	1.506 (1.083-2.313)	0.018*
ZNF334 methylation (high *vs* low)	1.491(1.006-2.102)	0.041*	1.391 (0.967-2.002)	0.076

*P < 0.05, **P < 0.01.

### GSEA of Prognosis-Related MeDEGs

**Figure 7:**
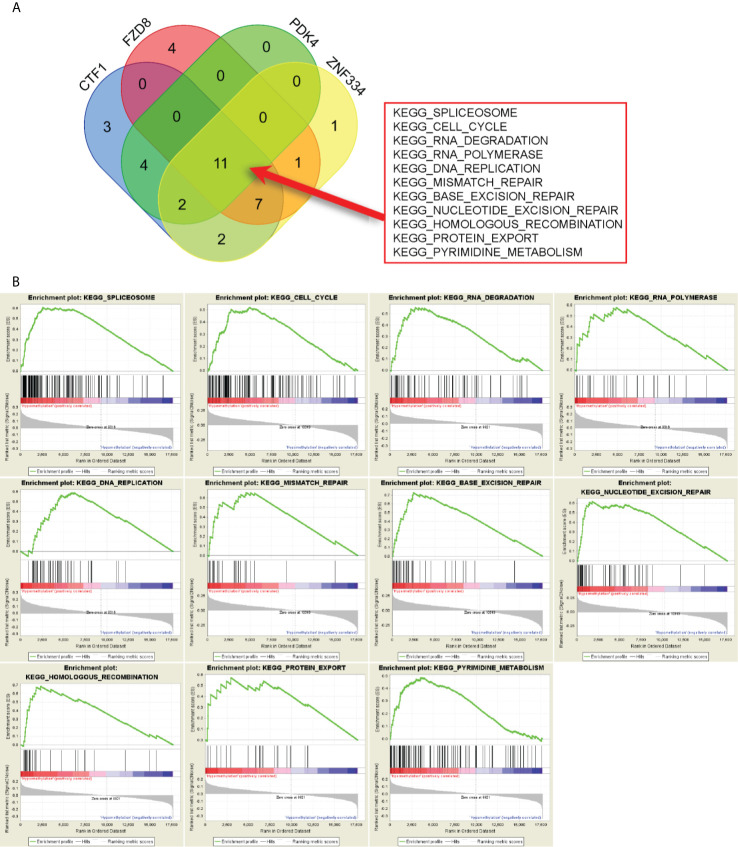
Gene set enrichment analysis (GSEA) of prognosis-related MeDEGs. **(A)** Intersection of pathways enriched by hypermethylation of CTF1, FZD8, PDK4, and ZNF334 is shown in the diagram. **(B)** GSEA of PDK4 is shown as example.

GSEA results revealed the potential mechanisms of prognosis-related MeDEGs. A total of 11 consensus terms were obtained from the enriched KEGG terms and included “Spliceosome”, “Cell cycle”, “RNA degradation”, “RNA polymerase”, “DNA replication”, “Mismatch repair”, “Base excision repair”, “Nucleotide excision repair”, “Homologous recombination”, “Protein export”, and “Pyrimidine metabolism” (**Figure 7A**). PDK4 enrichment is represented as an example in **Figure 7B**.

## Discussion

Accumulating evidence has indicated that aberrant DNA methylation modification is a critical molecular event in HCC progression. Hypermethylated status in the promoter of tumor suppressor genes (13), cyclin (14), and DNA mismatch genes (15) has been studied in depth. HCC cell methylation profiles have become a new field of tumor biomarker study (16). Furthermore, DNA methylation has been recognized as a potential therapeutic target due to its reversibility (17). Therefore, MeDEG identification will provide more information on the role of methylation in HCC.

Bioinformatics analysis in the present study resulted in 81 MeDEGs. GO analysis indicated that the main terms are related to transcription dysregulation. For instance, Kruppel-like factor 4 (KLF4) has been identified as a transcription factor that can suppress the expression of Ring1- and YY1-binding protein and inhibit HCC tumorigenesis (18). Moreover, KLF4 expression was epigenetically inhibited by CpG-island hypermethylation (19). Therefore, it was speculated that methylation can indirectly control gene expression by regulating transcription factors. The present study also identified tumor-associated calcium signal transducer 2 (TACSTD2) as a MeDEG, which has been reported to be down-regulated in primary HCC tissue. However, no research has demonstrated that TACSTD2 is regulated by methylation in HCC, which will be the subject of future studies. KEGG pathway analysis further defined the role of MeDEGs in HCC. It is noteworthy that “Pathways in cancer” was the most enriched pathway in which Ras-related C3 botulinum toxin substrate 2 (RAC2), guanine nucleotide-binding protein subunit beta-4 (GNB4), prostaglandin E2 receptor EP4 subtype (PTGER4), G1/S-specific cyclin-E2 (CCNE2), Frizzled-8 (FZD8), transforming growth factor beta-3 (TGFB3), laminin subunit alpha-2 (LAMA2), glutathione S-transferase Mu 1 (GSTM1), and matrix metalloproteinase-2 (MMP2) are involved. Though RAC2 (20), GNB4 (21), PTGER4 (22, 23), CCNE2 (24), FZD8 (25), TGFB3 (26), LAMA2 (27), and GSTM1 (28, 29) have been reported to be regulated by methylation in multiple cancers, very little is known about the regulatory mechanisms by which methylation is involved in HCC. Moreover, the term “Hepatocellular carcinoma” is enriched with FZD8, ribosomal protein S6 kinase alpha-6 (PRSAKA6), TGFB3 and GSTM1. FZD8 has been demonstrated to be an important cell membrane receptor that mediates the Wnt signaling pathway in HCC (30, 31). Feng et al. (32) have indicated that TGFB3 can function as a modulator to promote the metastatic phenotype of non-metastatic HCC cells induced by TGFB1. GSTM1 polymorphisms have been identified as biomarkers of HCC development and risk in different regions (33–37).

In addition, methylation status of cardiotrophin-1 (CTF1), FZD8, pyruvate dehydrogenase kinase 4 (PDK4), and ZNF334 was associated with overall survival. Similar results were obtained by performing conjoint analysis of methylation, expression, and prognosis. CTF1 is a mitogenic cytokine of the interleukin 6 family, which is a hepatocyte survival factor that is up-regulated during liver regeneration in animal models (38). Bustos et al. (39) have indicated that CTF1 prevented colon cancer cell proliferation in the liver depending on T and NK cells. However, the function and regulatory mechanism of CTF1 in HCC remains controversial. A recent study has indicated that prognosis-related PDK4 is down-regulated in HCC tissues, while PDK4 knockdown promotes HCC cell proliferation, migration, and invasion (40). Moreover, arsenic-induced silencing of PDK4 in hepatic cells is mediated by histone H3 lysine 9 methylation in the promoter (41). ZNF334 lymphocyte expression can be regulated by tumor necrosis factor α. However, little is known about ZNF334 in tumors (42). The present study first indicated that methylation of PDK4 and CTF1 is a potential independent biomarker for prognosis prediction. More studies are needed to verify this hypothesis.

GSEA clarified the mechanisms by which prognosis-related MeDEGs drive tumorigenesis. A total of 11 pathways that involved prognosis-related MeDEGs and the “spliceosome” pathway were the most significantly enriched. The spliceosome consists of five ribonucleoprotein subunits and protein cofactors and has been demonstrated as a critical and complicated mechanism in mRNA synthesis regulation of eukaryotic cells (43). Krogh et al. (44) have indicated that ribose methylation interrupts snRNA interactions and affects the splicing process in a T cell leukemia model. According to the GSEA results, almost every step of gene transcription and translation is enriched by methylation of prognosis-related MeDEGs.

The present study has some limitations. Firstly, the present research is mainly based on bioinformatic analysis of TCGA database and verification of identified genes and pathways is insufficient. Secondly, it is generally known that microsatellite-instability (MSI) is associated with aberrant methylation in HCC. Illumina Human Methylation 450K BeadChip data analyzed in the present research did not supply any MSI information. Thus, it is difficult to reveal the relationship between MSI, methylation and prognosis and more validation experiments are needed in the future.

In conclusion, MeDEGs were identified by analyzing the expression profiles and methylation data of HCC samples from TCGA database. GO and KEGG pathways analyses verified the MeDEG mechanisms. Furthermore, four prognosis-related MeDEGs and methylation status of PDK4 and CTF1 were identified as potential biomarkers for survival prediction and treatment.

## Data Availability Statement

The original contributions presented in the study are included in the article/supplementary material. Further inquiries can be directed to the corresponding author.

## Author Contributions

H-MY conducted the study. YL, BM, and PJ applied the experiments on TCGA project. YL wrote the manuscript. All authors contributed to the article and approved the submitted version.

## Funding

The study was funded by the Revitalizing Liaoning Talents Program (grant number XLYC1907004).

## Conflict of Interest

The authors declare that the research was conducted in the absence of any commercial or financial relationships that could be construed as a potential conflict of interest.

## Abbreviations

HCC, Hepatocellular carcinoma; CpG, Cytosine-phosphate-guanine; MeDEGs, Methylation-regulated differentially expressed genes; TCGA, The Cancer Genome Atlas; GSEA, Gene set enrichment analysis; DEGs, Differentially expressed genes; DMGs, Differentially methylated genes; FC, Fold change; GO, Gene ontology; KEGG, Kyoto Encyclopedia of Genes and Genomes; Hyper-LG, hypermethylation and low-expression MeDEG; Hypo-HG, hypomethylation and high-expression MeDEG; ZNF334, Zinc finger protein 334; CTF1, Cardiotrophin-1; FZD8, Frizzled-8; PDK4, Pyruvate dehydrogenase kinase 4; KLF4, Kruppel-like factor 4; TACSTD2, Tumor-associated calcium signal transducer 2; RAC2, Ras-related C3 botulinum toxin substrate 2; GNB4, Guanine nucleotide-binding protein subunit beta-4; PTGER4, Prostaglandin E2 receptor EP4 subtype; CCNE2, G1/S-specific cyclin-E2; TGFB3, transforming growth factor beta-3; PRSAKA6, Ribosomal protein S6 kinase alpha-6; LAMA2, Laminin subunit alpha-2; GSTM1, Glutathione S-transferase Mu 1; MSI, Microsatellite-instability.
